# Self-limited childhood epilepsies are disorders of the perisylvian communication system, carrying the risk of progress to epileptic encephalopathies—Critical review

**DOI:** 10.3389/fneur.2023.1092244

**Published:** 2023-06-14

**Authors:** Péter Halász, Anna Szũcs

**Affiliations:** ^1^Department of Neurology, University Medical School, Pécs, Hungary; ^2^Institute of Behavioral Sciences, Semmelweis University, Budapest, Hungary

**Keywords:** self-limited epilepsy spectrum, centrotemporal spike, epileptic transformation of system networks, electrical status epilepticus in sleep, epilepsy progress, role of NREM graphoelements

## Abstract

“*Sleep plasticity is a double-edged sword: a powerful machinery of neural build-up, with a risk to epileptic derailment*.”

We aimed to review the types of self-limited focal epilepsies...“i.e. keep as two separate paragraphs” We aimed to review the types of self-limited focal epilepsies: (1) self-limited focal childhood epilepsy with centrotemporal spikes, (2) atypical Rolandic epilepsy, and (3) electrical status epilepticus in sleep with mental consequences, including Landau–Kleffner-type acquired aphasia, showing their spectral relationship and discussing the debated topics. Our endeavor is to support the system epilepsy concept in this group of epilepsies, using them as models for epileptogenesis in general. The spectral continuity of the involved conditions is evidenced by several features: language impairment, the overarching presence of centrotemporal spikes and ripples (with changing electromorphology across the spectrum), the essential timely and spatial independence of interictal epileptic discharges from seizures, NREM sleep relatedness, and the existence of the intermediate-severity “atypical” forms. These epilepsies might be the consequences of a genetically determined transitory developmental failure, reflected by widespread neuropsychological symptoms originating from the perisylvian network that have distinct time and space relations from secondary epilepsy itself. The involved epilepsies carry the risk of progression to severe, potentially irreversible encephalopathic forms.

## Introduction

We have focused just on a part of the perisylvian spectrum (1) involving non-epileptic forms where centrotemporal spikes (CTS, Rolandic spikes) occur without a ripple crown; self-limited focal childhood epilepsy with centrotemporal spikes (SeLECTS, Rolandic epilepsy—RE); benign centrotemporal epilepsy with CTS (BETCS); atypical SeLECTS; and developmental and/or epileptic encephalopathy with spike-wave activation in sleep (EE-SWAS) as electrical status epilepsy in sleep and Landau–Kleffner syndrome (ESES/LKS).

Reviewing the members of SeLFE, the spectral continuity of SeLECTS to ESES/LKS is less clear in the rest of the group. Panayiotopoulos syndrome (PS) is an autonomic epilepsy, with strong occipital interictal epileptiform discharges (IEDs) and a vague seizure onset zone that is not typically centrotemporal; in addition, it seems to have distinct genetic bases (2). In Gastaut-type late-childhood occipital epilepsy (GOE) and photosensitive focal occipital epilepsy (POE), there is little risk of encephalopathic transformation; rather, POE has been described as overlapping with or transforming into idiopathic generalized epilepsy (3).

Our endeavor is to support the hypothesis that epilepsies build upon functional brain systems and that there is a spectral continuity in the perisylvian epilepsy spectrum (PE).

## Changing interpretations of the spectrum

SeLFE accounts for about 20–25% of childhood epilepsies. Originally, this included three types (RE, PS, and GOE), but the recent ILAE classification involved a fourth member, POE. Those epilepsies can mutually transform into each other and occur in a common age window (4). The age of onset is between 3 and 10 years, with a peak around 6–7 years in 90% of cases, and recovery occurs before the age of 15–16 years (5).

Challenging the classic definition of epilepsy, in which the presence of seizures is a cornerstone, these epilepsies do not necessarily manifest seizures, or just rarely, and the neuro-psychiatric harm may originate from interictal discharges in NREM sleep and not exclusively from seizures. Patients have typically < 10 seizures, and 10–20% of them present with just one (6). Most seizures evolve during NREM sleep or drowsiness; the probability of awake seizures is < 10% (7, 8). Rolandic (centrotemporal) spikes (CTS) typically accumulate during NREM sleep (1). It has been reported that 1.2–3.5% of normal healthy children between 5 and 12 years of age (9, 10) and 6–34% of siblings and relatives of patients carry CTS (11–13), CTS in SeLECTS are special in several ways: they are not necessarily concordant with the seizure onset zone, and most interestingly, they have a shifting, vague localization that moves widely around the perisylvian regions, even bilaterally. Based on these features, including a hint for the genetic inclination to epilepsy, Panayiotopoulos named RE, PS, and GOE “genetically determined seizure susceptibility syndromes” (14).

In the 1970's, Patry et al. (15) recognized a peculiar devastating condition characterized by a tsunami of spikes flooding both hemispheres during more than 85% of NREM sleep. It presented with sparse seizures, a global mental regression, and variable psychiatric symptoms, depending on the duration of the period spent with abundant sleep spiking (16). Waking stopped the flood of discharges immediately. This condition has been more or less drug-resistant to traditional anti-seizure medication (ASM), while steroid treatment has been more effective. It proved to be self-limiting, but about 50% of patients were left with variable degree irreversible cognitive loss (17). Tassinari named the condition “electrical status epilepticus in sleep” (ESES), or “Penelope syndrome,” referring to the Odysseus legend (18).

A similar condition called acquired epileptic aphasia had been described earlier by Landau and Kleffner (19). In this condition, the frequent epileptic spikes accumulated in the posterior temporo-parietal region of the dominant hemisphere, around the Wernicke area, propagating to the adjacent regions, often involving homolog areas. The NREM sleep-related abundancy was like that of ESES, only showing a focal dominance.

It seemed that the cognitive symptoms were related to interictal epileptic discharges (IEDs) without the participation of seizures (16, 20).

In exploring the history ESES/LKS patients, it was found that early perinatal or infantile injuries had occurred in more than half of cases (21), and in the rest, one of SeLFE had preceded ESES/LKS. This was congruent with the fact that CTS was present in the waking EEG of those malignant forms.

The seemingly distinct “benign” and encephalopathic syndromes have slowly gotten close to each other, as evidenced by variable severity language and other cognitive impairments detected in the benign forms (22–44). Now, the ILEA also has accepted cognitive impairment as a standard symptom of SeLFE (45).

The gap between the two groups has decreased also due to the more permissive cutoff percentage of sleep covered by epileptic discharges (46). If the “spike-wave index” in sleep reaches 40–50% and there is a mental regression, ESES diagnosis is considered. This 40–50% ratio is close to what CTS can reach in the sleep EEG of the “benign” forms. Thus, both the continual presence of sleep enhancement of discharges and cognitive impairment—the two in a close positive relationship—have supported the spectrum concept.

The recognition of “atypical Rolandic epilepsy” as an intermediate variant has brought us closer to understanding its progress to the encephalopathic forms. Those atypical forms are characterized by early seizures, peculiar EEG abnormalities, and poor neuropsychological outcome. First, Aicardi and Chewrie gave an account of the atypical features (47): myoclonic and atonic seizures and spike-wave runs in sleep and learning difficulties. Doose named the condition the pseudo-Lennox syndrome (48). We issued a unified concept about the self-limited syndrome spectrum in the form of the system epilepsy of the perisylvian human language network (PN) (1). Fejerman summarized the warning signs forecasting encephalopathic progression in 43 children (49). The warning features included long atypical seizures, oro-motor status epilepticus, and an early cognitive loss more important than in the “benign” forms, as well as spike-wave runs, frequent CTS, and an abnormal EEG background. In the following years, several papers confirmed these findings (4, 50–53).

In an EEG-fMRI study on atypical SELECTS (54), distant cortical and subcortical BOLD changes were associated with continuous spiking in NREM sleep. The group analysis revealed a thalamic activation. In another study, spike dipoles predicted the atypical outcome in RE; the source dipoles were situated more posteriorly than in the typical group, signaling an atypical course (55).

In summary, the atypical forms of SELECTS manifest more severe epileptic and cognitive outcomes compared to the classical ones. It is unknown whether these atypical forms represent transit stops toward the encephalopathic forms or a genetically distinct variant. Long-term follow-up studies are needed for a better understanding.

## Genetic background

The family aggregation and the contribution of an inherited risk to develop SeLFE are known (56). In addition to revealing hidden links and mechanisms with other conditions, genetic research has provided data on the spectral togetherness of SeLFE and EE-SWAS, also called the epilepsy–aphasia spectrum (EAS) (57).

SELECTS and its atypical form have a polygenic, complex trait of inheritance (58–63).

The most established mutations underlying EAS are the pathogenic variants of the GRIN2A gene (coding the N2 unit of the NMDA receptor) (64, 65). GRIN2A is associated with a broad spectrum of childhood epilepsies including generalized epilepsy with febrile seizures, SELECTS, and PS, representing complex genotype–phenotype variations (66). The GRIN2A-FOXP2-SRPX2/uPAR signal network might be related to the development of the shared, though variable, severity language deficit in the spectrum. Thus, both the epileptic discharging and genetic causes (with no epilepsy) might contribute to the neuropsychological impairment of children with EAS conditions (67).

In addition to the common genetic traits of SELECTS, such as the atypical form, the latter variant may have a distinct genetic background too: PRRT2 mutations and several additional genes have also been linked to it (68).

The SCN1A genes (coding for the voltage-gated Na+ channel alpha subunit NaV1.1), which form the most relevant epilepsy gene family, appear to be associated with the outcome but not the development of the Panayiotopoulos syndrome (69).

Besides several epileptic and non-epileptic conditions, SCN1A gene mutations link with autism spectrum disorder, offering a genetic base for the spectral belonging of autism spectrum disorder to SeLFE (70).

Moreover, the spike distribution in SeLFE has a genetic base (71), and interestingly, CTS without epilepsy follows a highly penetrant autosomal dominant inheritance pattern with an equal sex ratio in the epilepsy families. The CTS locus might act in combination with one or more loci to produce the phenotype of RE (72–74).

In summary, SeLFE, including the malignant end, seems to be underlain by multiple gene mutations. A single mutation may have different phenotypic manifestations and vice versa, and a phenotypic variant may be related to diverse genotypic alterations. It is likely that environmental factors also contribute to the development of those conditions. This complexity of factors may cause the overlap of different epilepsy types with each other and with non-epileptic conditions.

The EAS spectrum's most known and established gene mutation affects the GRIN2A gene, involving the development of epilepsies and language processing too.

## Cognitive impairment is an essential feature of SeLFE, the system epilepsy of the PN

Language research has revealed the enormous development of the perisylvian language area from chimpanzees to humans, the enlargement (75) being most remarkable in higher association cortices.

**Figure 1:**
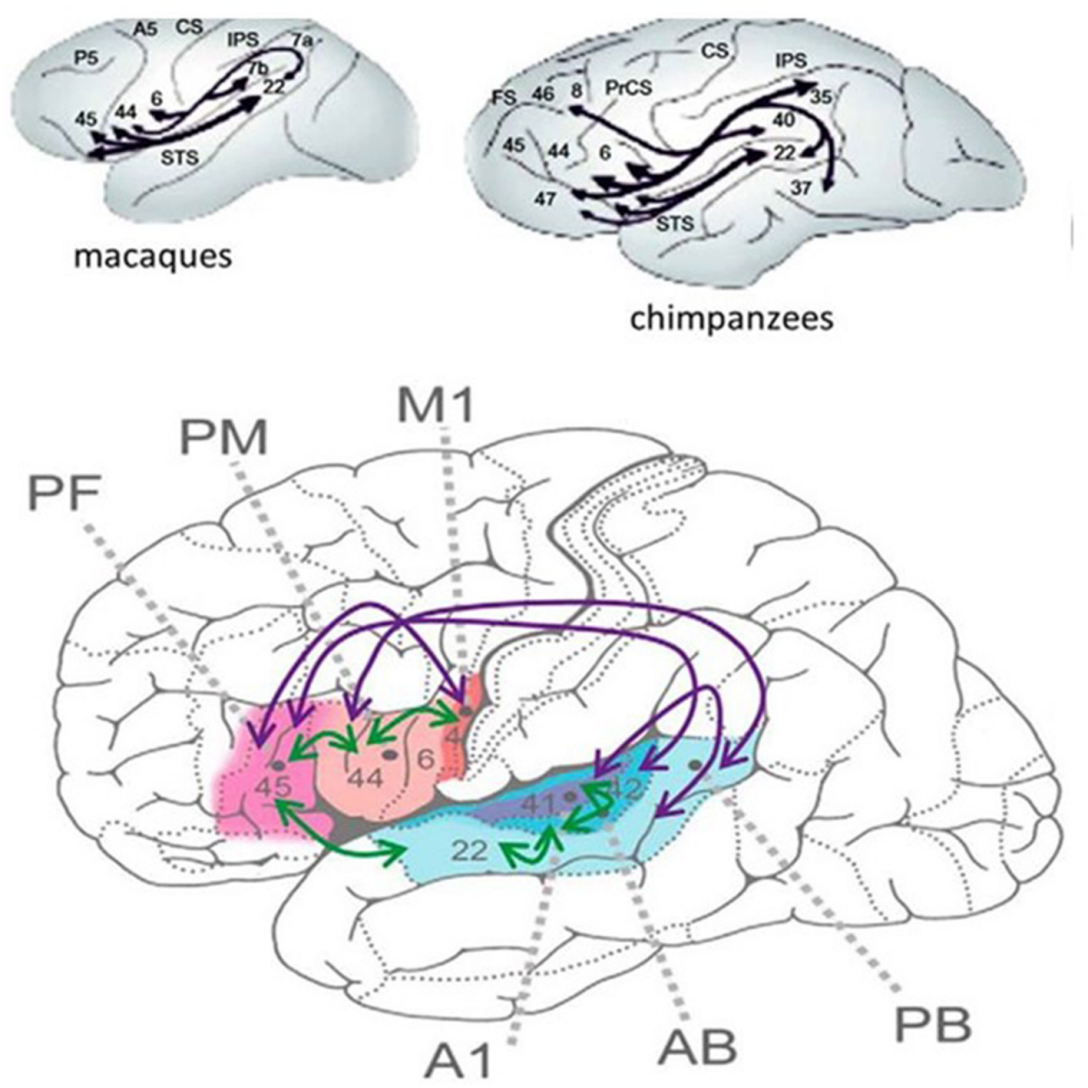
**(Bottom)** The human perisylvian language network [after Schomers et al. (75)]. M1 = primary motor area of speech expression (flesh -like-color), PM = premotor Broca area, PF = prefrontal area working memory (pink), A1, AB, and PB auditory areas. The purple arrows represent the interconnections among areas only existing in humans. Green arrows: interconnections only in animals. **(Top)** The interconnection of the perysylvian areas in apes. The numbers represent Brodmann areas. Black lines show interconnections.

The human language network interweaves the sensory, motor, auditory, and articulatory machinery and occupies the perisylvian cortex with premotor, anterior temporal, and temporo-occipital associative fields. The main information stream is organized through the dorsal and ventral pathways. The dorsal arcuate pathway, bi-directionally connecting the Broca and Wernicke areas, is involved in executive and comprehensive language functions. This bundle has importantly increased during human evolution, allowing the faster transmission of information (Figure 1).

The PN, widely covering the frontal–prefrontal temporal and temporo-occipital cortices, deserves the name “human communication system” for its key roles in speaking, reading, and writing (76). The circumscribed epileptic symptoms of SELECTS are associated with a much wider cognitive syndrome, which may precede and be unrelated to clinical and EEG signs, building upon them later (77).

It is possible that the widespread neuropsychological symptoms and related neuroimaging changes preceding the onset of epilepsy mark the long suspected developmental failure or delay underlying SeLFE. The concept of system epilepsy traces epilepsies back to a derailment of physiological brain functions (1, 78–81), i.e., epilepsies manifest the disfigured features of the hosting brain systems, as seen in this epilepsy spectrum too.

## Changes of hemispheric dominance in the SeLFE

The cognitive symptoms of SELECTS have exceeded the localization of epilepsy limited to the intrasylvian part of the somatosensory strip. SELECTS may be a developmental disorder (82, 83), and its impact on hemispheric specialization has been long suspected. The participation of hemispheric work-share in the human cognitive functions is an important and hitherto under-reported issue (84). The recognition of the changes in the hemispheric cooperation in SeLFE patients shed new light on the cognitive impairment issues in the spectrum. In a study on 27 SELECTS children compared with healthy controls (85), Datta et al. (85) found that the language reorganization in SELECTS patients took place in bilateral or right hemispheric language networks, with a strong focus in anterior language regions. These functional changes can be interpreted as important compensatory strategies of the central nervous system to stabilize cognitive, especially language performance.” A recent study confirmed the alterations of the hemispheric specialization in SeLECTS children (86). Thus, SeLECTS can provide a valuable model for studying the plastic self-reconstruction of communication-related functions, thus allowing to develop better language rehabilitation approaches.

## Changes of CTS across the spectrum reflect the transitions and severity of conditions

CTS is a high amplitude sharp-and-slow wave complex, appearing typically in the centrotemporal region and around. The “spikes” are triphasic sharp waves of 100–300 microvolt amplitude, rather more consistent with sharp waves than spikes. They include an initial low-amplitude positivity followed by a high-amplitude negativity and another low-amplitude positivity. The amplitude of the closing slow wave is lower than in the spike-wave pattern of absence epilepsies. The discharges may be singular or occur in doublets and triplets; they can be unilateral, bilateral, or multifocal even in one recording. Their timely distribution ranges from rare random (1–3/10 s) to frequent or even continuous, appearing in runs. The amplitude map shows characteristic frontal positive and temporal negative tangential dipoles with an axis perpendicular to the Sylvian fissure (87). Source imaging studies have localized it to the lower (intra-Sylvian) part of the somatosensory cortex (88). CTS without ripples during NREM sleep has been found in 2–4% of healthy children and in childhood mental health conditions such as autism spectrum disorders (89, 90) and attention-deficit/hyperactivity disorder (ADHD) (91–93).

These CTSs emerge in the time window of SELECTS. Fifteen percent of SELECTS children's siblings had seizures and CTS (14), while 19% had CTS but no seizures. In a 5.5-years follow-up study on 40 children with non-epileptic disturbances and CTS on the EEG; only two patients had developed SELECTS, and in most cases, CTS disappeared without any clinical signs.

**Figure 2:**
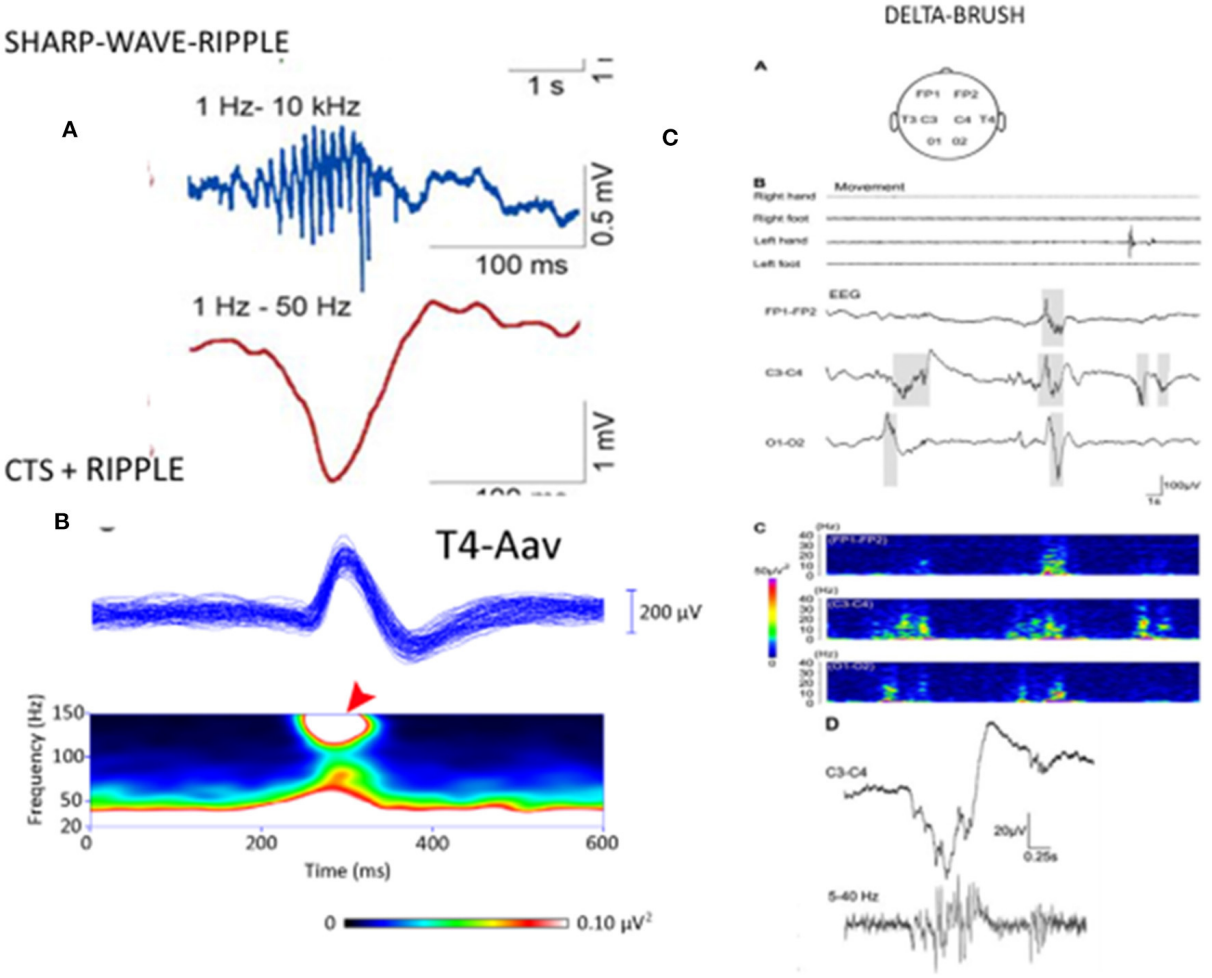
**[Left; (A)]** The sharp-wave ripple of the hippocampus: first line: ripple component. second line sharp wave component. **(B)** Centrotemporal spike (CTS)-ripple. First line: averaged CTS, second line spectrogram of the ripple component (red arrow). **(Right)** “Delta-brushes” and their spectrogram (on the bottom line enlarged) evoked by jerky newborn movements over the central region (C3-C4) on the EEG, **(A–D)** suggested to play a role in the development of the somatotopic representation of the newborn [after Milh et al. (97)]. Note the similarity of sharp-wave-ripple, CTS-ripple, and delta brush. Adapted with permission of Oxford University Press (97).

Several papers have reported the existence of high-frequency (80–250 Hz) oscillations (HFO) as a “crown” on the top of CTS (52, 94–96). van Klink et al. (94) found correlations between HFO (ripple) parameters and clinical severity, with the number of ripples, unlike spikes, showing a significant positive correlation with the number of seizures. CTS without HFO presented no seizures. The presence of more than two ripples predicted seizures, and more ripples correlated with poorer outcomes. In the encephalopathic variants (EE-SWAS), the abundant high-amplitude irregular spike and slow waves during REM sleep were associated with HFO (52, 93–96). In summary, CTS and HFO parameters signal the severity of syndromes in the spectrum (Figure 2). HFO associate with CTS in SELECTS, and there are more HFO in atypical SELECTS. Furthermore, when EE-SWAS evolves, the number, voltage, and amplitude of ripples augment even further. This sequence is congruent with our recent knowledge about HFO as the best markers of epileptogenicity (98).

## CTS analog EEG patterns carrying developmental potential

**Figure 3:**
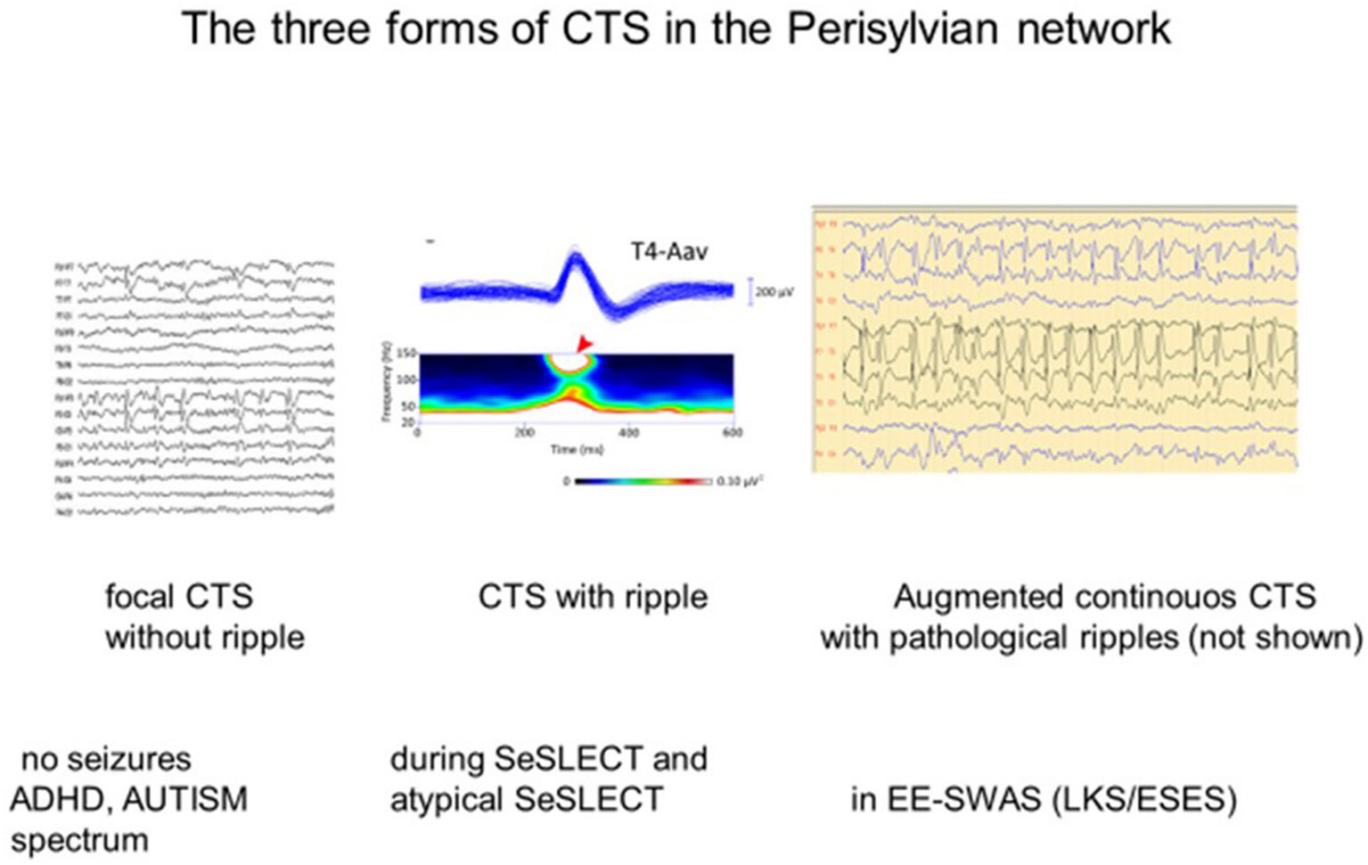
The three forms of the centro-temporal spike (CTS) across the self-limited focal childhood epilepsy spectrum. **(Left)** CTS without epileptic seizures; **(middle)** Rolandic epilepsy with ripples on the top of CTS [spectrogram; (top): averaged spikes, (middle): spectral frequencies on the top of spikes (red arrow)]. **(Right)** ESES discharge pattern assumed to be augmented CTS with pathological ripples (not shown) evidenced by Kobayashi et al. (94). Adapted with permission of Wiley Online Library (94).

A premature infant's “delta-brush” pattern (short fast spindle-like components coupling with a slow wave) is shown to mark the brain maturation of the preterm infant (97). It resembles CTS in several ways: morphology, suggested involvement in brain development and plasticity in a self-limiting age window, and sleep relatedness (Figure 3). Delta brush is the dominant pattern in premature infants *in utero* (99). Its voltage and density diminish by 9 months gestation and can be triggered by sensory stimuli in the same circuits that initiate spindles in the adult brain (100). There is an analog pattern in rodents, the “spindle brush” that has proved to be a model of delta brush (101). Spinal cord transection has been shown to decrease the activity of spindle brush; however, an external stimulus was not necessary for its initiation. Spindle brush was suggested to provide sensory feedback to the developing somatosensory and motor cortices serving the development of somatosensory cortical maps. It was suggested that both spindle brush and delta brush may promote adaptive plasticity in synaptic connections (100).

Sharp-wave ripple (SPW-R), the key element of hippocampal declarative memory consolidation, is another analog of CTS (101). During the epileptic transformation of the hippocampo-frontal declarative memory system, it turns to a useless pathological spike-fast ripple complex unable to process engrams (101, 102). These peculiar EEG patterns may mark a developmental function with the risk of derailment toward epileptic hyperexcitation. One may speculate that CTS without a ripple crown may represent an epileptic susceptibility disappearing before adolescence in most cases. When SELECTS develops and EE-SWAS manifests high-voltage continuous spikes, abundant pathological HFO increase in sleep (94).

## CTS, sleep spindling, cognitive impairment, and neuroimaging alterations

### CTS and sleep spindling

The relationship between spindles and IEDs, including CTS, remains contradictory in several aspects. While CTS reflects an increase of cortical excitability, numerous studies have pointed to the role of the thalamocortical system in its mechanism. Unlike most epilepsies where IEDs couple with CAP A1 slow waves, IEDs of SELECTS link with sleep spindles (103–106). This seems to contrast with another finding (107) on significantly lower mean values of the amplitude, duration, and density of sleep spindles in 30 typical SELECTS children, compared to age-matched controls. Consequently, in a cohort of SeLFE children's sleep EEG, a transient deficit of spindling was shown in the 12–15 Hz range, and the spindle rate was anti-correlating with the spike rates. It was the spindle rate and not the spike rate that predicted cognitive performance. This finding was interpreted as a support for focal thalamocortical dysfunction related to spindles (108).

Another study (109) demonstrated that auditory stimulation during sleep suppressed CTS (possibly by inducing refractoriness in the shared thalamocortical network) and evoked sleep spindles. This finding suggests a rivalry between the two patterns within the thalamocortical system.

The interference of spikes and spindles was shown in other epilepsies as well; for example, the decrease of hippocampal spindles was shown to be proportional with the rate of hippocampal spikes during NREM sleep in mesio-temporal lobe epilepsy (110). In 1978, Gloor made a similar observation about the assumed inverse relation of the spindles and spike-wave discharges (SW) in absence epilepsy.

Another study investigated the coupling of hippocampal spikes with temporal spindles registered by foramen ovale electrodes. Those spindles that coupled with spikes had altered morphological features, and the spindle alterations seemed to interfere with the memory process.

Dahal et al. (111) have demonstrated that “IEDs functionally interact with diverse and remote cortical regions in the human brain via induction of coupled physiological spindles.” The distribution of IED-spindle coupling was patient-specific but consistently localized outside of the seizure onset zone. Spindles coupled to IEDs were consistently found in brain regions that had not generated IEDs and seizures. These regions were unlikely to be recruited into early stages of seizure propagation. Therefore, the “expression of spindles temporarily locked to IEDs likely reflects a directional interaction from the epileptic to the non-epileptic zone.” The altered spindle properties identify brain regions with interictal network dysfunctions that could possibly contribute to cognitive co-morbidities and disease progression. The induction of spindling by IEDs may serve the spread of the epileptic network. The Dahal study asserts that the IEDs' coupling with spindles may reflect two kinds of relationships: one is the anticorrelation of the spikes at the expense of spindles (and cognition?); the other is the fueling of the epilepsy process (111). It is notable that the IEDs in the Dahal study were not related “ab ovo” to spindles, as is the case in self-limited epilepsies (103–106). The spindle-inducing potential of IEDs has not been tested in SeLFE patients.

### Spindles, CTS, and cognition

The role of spindles in organizing cognitive functions has been evidenced by several experimental and clinical studies both in children and adults (100); however, the question of whether spindles have a direct or indirect impact has remained unanswered.

Animal studies in the rat somatosensory cortex (112) and visual cortex (113) have asserted that IEDs' harm on declarative memory and cognition is actually due to their interference with spindles in certain epilepsies (99).

Vaudano et al. (114) studied 8,950 CTSs in 23 SELECTS patients, finding hemodynamic changes within the language network and in the bilateral insular cortices. CTS frequency, even in wakefulness, interfered with the functioning of the language networks.

Disturbances seemingly caused by epilepsy in EE-SWAS may result from an impairment of the sleep homeostatic functions, as shown by Bölsterli et al. (115, 116); the abundant sleep discharging in EE-SWAS interfered with the downscaling of sleep slow oscillation, thus obstructing synaptic refreshment.

From a theoretical point of view, this means that cognitive impairment might be an essential consequence of epilepsies, originating in the epilepsy–sleep–cognition interrelations (80).

Those studies on the participation of the thalamocortical system in the pathomechanism SeLFE provide new meanings to the role of early thalamic hemorrhages involving the reticular nucleus (NRT). In 90% of children with ESES and a history of thalamic hemorrhage, the NRT was affected (117); this is congruent with the notion that the NRT provides a GABAergic recurrent inhibition to thalamocortical sleep oscillations, regulating the rhythmicity from physiologic spindling to epileptic spike and wave discharges (118).

### Imaging and CTS

New imaging methods have allowed to reveal widespread connectivity disorders underlying CTS in subcortical structures such as the putamen (119–124). Neuroimaging studies have evidenced CTS's harm on language and thalamocortical networks even without epilepsy (120). A 2-year follow-up neurocognitive MRI study of 8–15-year-old SELECTS children and 41 matched healthy controls (122) revealed that SELECTS children had poorer cognitive performance and a thinner cortex in widespread regions, remaining unchanged across the follow-up period. They had putaminal abnormalities both at the baseline and during follow-up. Cortical thickening–thinning of SELECTS patients and controls moved in opposing directions. In patients, thinning involved a small part of the prefrontal cortex, while a larger part of the cortices thickened. This study demonstrated an “ab ovo” presence of cognitive drawback in SeLECTS children, while cortical and putaminal abnormalities started after the beginning of epilepsy. Other works have confirmed that the language network impairment precedes epilepsy (125).

The results of the work of Bourel-Ponchel et al. (124) supported the idea that disorganizations of the CTS-related neuronal network may disrupt local and distant networks with a possible impact on functional and maturational processes. They demonstrated the desynchronization and disorganization in distant networks' functional structures several 100 ms before each IED, pointing to how chronic impairments may develop.

## The overlap of SELECTS and absence epilepsy

The simultaneous presence of absence epilepsy and SeLFE in children is a special issue (126, 127) suggesting a shared network background.

Huguenard (128) wrote an interesting paper about the overlapping circuit features of the centro-temporal spikes and SW of absence epilepsy.

In the thalamocortical system, the GABAergic output from the NRT regulates the recurrent thalamo-cortical inhibitory oscillations. The reciprocal interconnections between NRT cells normally serve to desynchronize thalamocortical activity and prevent generalized activity. Up or downregulation of intra-NRT inhibition has been evidenced to suppress or promote intrathalamic epileptiform activity, respectively (129, 130).

Small fractions of isolated thalamic slices from a β3 mouse mutant were found to produce a reverberant activity that resembled absence seizures, remaining restricted to small sectors of the slice. The conclusion was that “at least in the isolated thalamus, circuit mechanisms exist for the production of local recurrent oscillatory responses and may produce a focal thalamocortical spike.”

This raised the possibility of two types—sectorial and wide-spread, bilateral—reverberating absence-like activities in the thalamocortical system, at least in isolated slices. Thus, the existence of a common circuit underpinning CTS and spike-wave discharges was suggested. This concept offers a shared pathomechanism of the two epileptic patterns in a common time window of childhood, together with recent genetic studies confirming their overlap (127).

## The electrophysiological mechanism of the transition from SELECTS to the encephalopathic forms (EE-SWAS)

SELECTS may progress to EE-SWAS in <10% of cases (4), but there are no data about the transformation ratio to atypical SELECTS. It resembles the encephalopathic forms in manifesting poorer neuropsychological performance and higher frequencies/densities of HFO. Moreover, neuroimaging studies put the atypical cases closer to the encephalopathic variants (53, 54).

The electrophysiological mechanism of the transformation from SeLECTS to EE-SWAS is not understood. Clinically, a progress from benign epilepsies to severe or tragic encephalopathies with functional losses (global mental decline or acquired aphasia/psychiatric symptoms) can be seen. In the EEG, there is an extreme augmentation of IEDs with high-voltage continuous spiking, slow waves, and high-frequency pathological ripples showing a variable extent of secondary bilateral synchrony.

The transformation strongly links with NREM sleep, while awakening abolishes those EEG signs. After a critical time (~18 months), the cognitive loss becomes irreversible.

The suggestions for pathomechanism are based on the assumed pathophysiologic features and nomenclature (continuous spike waves during slow-wave sleep—CSWS) that still reflect the old bilateral primary spike-wave synchronization concept, which is outdated now even for genetic generalized childhood absence epilepsy.

Concerning nomenclature, it is confusing that CSWS (an EEG term) is still widely used for naming a clinical syndrome, while the ESES name (another EEG term) stands for an EEG pattern plus encephalopathy.

The supposed notion of “primary bilateral synchrony” is in sharp contrast with all the neurophysiological studies that demonstrate unihemispheric secondary synchronization with propagation through the corpus callosum from one hemisphere to the other with a time delay (30–40 ms) and with a leading hemisphere of epileptic discharges (20, 131). The focal concept is supported by several additional features: the building up and down of IEDs at the onset and at the end of the active ESES period, the ictal semiology, and focal IEDS in waking. Successful surgical interventions, especially the multiple subpial transections (132, 133) in LKS children, support the focal origin of IEDS too. The unihemispheric phase reversal usually points to the centrotemporal region. Our preliminary mapping studies have revealed that the IEDs and the ESES discharges showed similar unihemispheric averaged spike voltage maps with a perpendicular orientation to the Sylvian fissure (21).

In accordance with the above findings, we assume that the electrical pattern of ESES/LKS is an augmentation of CTS discharges, but this assumption warrants further studies in the future.

## Discussion

Exploring the SeLFE spectrum, we are witnessing important conceptual changes.

Firstly, these epilepsies used to be considered harmless; now, a variable degree neuropsychological impairment shared by the involved conditions is recognized.

Secondly, we can now identify the hosting brain system, the PN, as highlighted by neuroimaging and neuropsychological studies. Therefore, we suggest that the SeLFE spectrum fulfills the criteria of system epilepsy.

It seems confusing that CTS and seizures localize to the intra-Sylvian somatosensory cortex, while neuropsychology symptoms cover, as confirmed by fMRI studies, much wider regions. Those findings revealing the presence of cognitive symptoms before epilepsy onset seem to offer a clue: SeLFE might emerge in a developmentally compromised brain system (77).

There are contradictory data in the literature on the relationship between IEDs and sleep spindles. Some researchers have found a spindle suppressing effect of IEDs (107, 108, 134), while others have shown an anticorrelation of IEDs and spindles, assuming their rivalry in the thalamocortical system (108–110), and certain authors have found that IEDs coupled or even induced spindles (111, 135).

The Parma school demonstrated in several papers (136) the coupling of IEDs with CAP A1 slow waves in most epilepsies, and this has been further confirmed (110). At the same time, in the group of SeLFE, IEDs were associated with the gamma frequency, and this was interpreted as an association of IEDs with sleep spindling (103, 104, 106).

Since the link of sleep spindling with cognitive functions has been evidenced by several studies, the relationships of IEDs with spindles might explain the cognitive loss associated with epilepsy (100). It seems that the relationship of IEDs and spindles needs to be studied specifically, analyzing the IEDs of distinct epileptic networks.

SeLFE are special in that the cognitive impairment seems to precede clinical epilepsy. Thus, a secondary epileptic derailment of a handicapped PN system (due to genetic developmental abnormalities and evidenced by neuroimaging, e.g., early cortical thinning) is assumed (77).

Sleep slow waves and spindles are organized in strict boundaries, with spindles situating on the up-states of the biphasic membrane state (up and down) fluctuations. Therefore, when focusing on slow waves or spindles, the behavior of the pair—the coupled spindle or slow wave—needs to be considered; it is senseless to speak separately about IEDs with slow waves or spindles. Sleep slow oscillations have a central orchestrating role in organizing spindles and IEDs (137).

A third conceptual change relates to the rare progress of SELECTS to EE-SWAS (ESES/LKS) (4). It is theoretically interesting, as it offers a model of epileptic progression. It is practically important too, since we do not have real remedies against it; traditional ASM does not provide enough protection. We consider this transformation a clear-cut epileptic progress, evidenced by the continuity of symptoms across the spectrum. Atypical SELECT forms are notable in this regard, with EE-SWAS approaching SELECTS in several, intermediate severity features.

HFO seem to have a paramount role in the transition from the “benign” to the encephalopathic forms of the spectrum. Physiological ripples (140–200 Hz) serve cognitive neural functions. The recognition of pathological ripples (200–500 Hz) was one of the most meaningful events in the last decades of epileptology (96, 138). Pathological ripples have become the best biological markers of epileptogenicity and the most reliable parameters of the localization and extension of the seizure onset zone for epilepsy surgery. Congruently with the pathoplastic role of NREM sleep in most epilepsies, both physiological and pathological ripples are enhanced in NREM sleep.

Gulyás and Freund (139) studied an analog switch from SPW-R to spike and pathological HFP and showed the possibility that physiological HFO activity can turn pathological. While physiological HFO is primarily the result of phasic perisomatic inhibitory currents, pathological HFO are population spikes of partially synchronous, massively bursting, uninhibited pyramidal cells.

The experimental works of the Steriade school, and later of Timofeev and coworkers, have shown the origin of the normal brain functions' epileptic transformation by the derailment of the normal homeostatic sleep functions (140–142). This is the basis of our system epileptic concept (80) that seems to be valid also in PE. In the common process of epileptogenesis, the normal functioning derails to epileptic working modes, as also signaled by the HFO transformations. In the SeLFE spectrum, CTS may progress to CTS + HFO, further augmenting in the encephalopathic forms, while making the normal PN an epileptic system.

The transformation to epileptic encephalopathic forms with the EEG evolution of the ESES/LKS discharge pattern and the HFO component can be considered as an experiment of nature, with the promise to highlight the significance of pathological HFO as the common electrographic pattern of epileptogenesis.

## Conclusions

SeLFE syndromes (SELECTS, atypical SELECTS, and EE-SWAS) form a spectrum hosted by the PN, turning into an epileptic system.

The trans-spectral continuity of the involved conditions is evidenced by several features such as language impairment, the shared presence of CTS and HFO (with changing electromorphology across the spectrum), IEDs' essential timely and spatial independence from seizures, NREM sleep relatedness, and the existence of the intermediate severity “atypical” form.

This epilepsy spectrum may be the consequence of a genetically determined transitory developmental failure, with an inbuilt danger of progressing to severe and potentially irreversible forms.

We call attention to some early developmental promoters of synaptic plasticity. Each of them features a slow wave combined with spindles or ripples and emerges typically during NREM sleep. They usually disappear in childhood but carry the risk of derailing toward epileptic exaggeration instead.

We suggest a common electrophysiological mechanism of transition from normal to epileptic functioning also in this spectrum. This transition rarely occurs; however, it carries a great heuristic significance and devastating consequences for many children, considering the high prevalence of PE in childhood. A similar transformation is also seen in medial temporal lobe epilepsy, which is associated with a switch from physiological (140–200 Hz) to pathological (200–800 Hz) HFO (102, 138, 139). To understand these transitions, further studies are warranted, and there is a need to change the technique of EEG recording, such as higher sampling rates and proper filtering.

## Suggestions for future research

- Improving terminology by making it congruent, with no contradictions.
- Performing long-term studies to clarify the relationship between the neuropsychological and epileptic symptoms (seizures and EEG) in time, quality, and severity.
- Assessing the prevalence and process of transformation from typical and atypical SeLFE to ESES/LKS using high (above 2,000 Hz) sampling.
- Voltage- and frequency-mapping studies to establish the ESES/LKS discharges' time and space parameters.

## Author contributions

PH had the idea and the concept, this was elaborated together by both authors, AS and PH. All authors contributed to the article and approved the submitted version.
